# Correlation of Leptin in Patients With Type 2 Diabetes Mellitus

**DOI:** 10.7759/cureus.57667

**Published:** 2024-04-05

**Authors:** Kajol Manglani, Nabila Nowshin Anika, Dhriti Patel, Sharan Jhaveri, Chaithanya Avanthika, Sourav Sudan, Zainab Alimohamed, Kripa Tiwari

**Affiliations:** 1 Internal Medicine, MedStar Washington Hospital Center, Washington, USA; 2 Pediatric Surgery, Baylor College of Medicine, Houston, USA; 3 Medicine and Surgery, B.J. Medical College and Civil Hospital, Ahmedabad, IND; 4 Medicine and Surgery, Smt. Nathiba Hargovandas Lakhmichand Municipal Medical College, Gujarat University, Ahmedabad, IND; 5 Pediatrics, Icahn School of Medicine at Mount Sinai, Elmhurst Hospital Center, New York, USA; 6 Medicine and Surgery, Karnataka Institute of Medical Sciences, Hubballi, IND; 7 Internal Medicine, Government Medical College, Rajouri, Rajouri, IND; 8 Division of Research & Academic Affairs, Larkin Health System, South Miami, USA; 9 Internal Medicine, Maimonides Medical Center, New York, USA

**Keywords:** type 2 diabetes mellitus, leptin resistance, leptin adiponectin, lifestyle modification (lsm), appetite regulation, glucose metabolism, neuropeptide, serum leptin

## Abstract

The exponential increase in diabetes mellitus (DM) poses serious public health concerns. In this review, we focus on the role of leptin in type 2 DM. The peripheral actions of leptin consist of upregulating proinflammatory cytokines which play an important role in the pathogenesis of type 2 DM and insulin resistance. Moreover, leptin is known to inhibit insulin secretion and plays a significant role in insulin resistance in obesity and type 2 DM. A literature search was conducted on Medline, Cochrane, Embase, and Google Scholar for relevant articles published until December 2023. The following search strings and Medical Subject Headings (MeSH terms) were used: “Diabetes Mellitus,” “Leptin,” “NPY,” and “Biomarker.” This article aims to discuss the physiology of leptin in type 2 DM, its glucoregulatory actions, its relationship with appetite, the impact that various lifestyle modifications can have on leptin levels, and, finally, explore leptin as a potential target for various treatment strategies.

## Introduction and background

The first case of diabetes mellitus (DM) was documented in 1552 BCE by Egyptian physician Hesy-Ra. He listed several remedies on the Ebers Papyrus for “passing of too much urine.” Eventually, in the second century, Aertaeus, a Greek physician, coined the term “diabetes,” which in Greek means “to pass through.” In the 17th century, Thomas Willis came up with the term “mellitus,” which in Latin means honey, intending to describe the sweetness of the urine [1,2].

DM is a chronic metabolic disease characterized by polyuria, polydipsia, and weight loss. There are several categories of the disease, including type 1, type 2, gestational diabetes, neonatal diabetes, maturity-onset diabetes of the young, and secondary causes due to endocrinopathies, steroid use, etc. About 5% to 6% of all pregnant women suffer from gestational diabetes, many of whom eventually progress to having type 2 DM. Type 1 DM is responsible for 5% to 10% of people with diabetes, and some studies show that type 2 DM affects more than 90% of those with the disease [3,4].

In 1994, the Centers for Disease Control and Prevention’s (CDC) diabetes program announced that diabetes had reached the pinnacle of epidemic proportions; since then, it has been steadily on the rise, posing a serious public health concern [5]. According to the data collected by the World Health Organization, the number of people affected by DM worldwide has surpassed 535 million and is predicted to cross 700 million by 2045. The estimated death toll due to the disease in 2019 was reported to be 1.5 million [6]. This is in part due to the rapidly increasing incidence of obesity, especially in industrialized countries. Statistical analysis gathered by the CDC concludes that the prevalence of obesity in the United States was 42.4% in 2017-2018, which is an increase from 30.5% according to the data collected from 1999-2000 [7].

The CDC also apprised that childhood obesity poses a significant threat and that one in five children and adolescents in the United States are obese [5]. Such changes would inevitably lead to drastic consequences. Data collected from the National Diabetes Statistics Report (2017) indicates that 87.5% of adults with diabetes are either overweight or obese [8]. Studies also show women with a body mass index (BMI) of 30 kg/m^2^ are at 28 times greater risk of developing diabetes than women with normal weight. With a BMI of 35 kg/m^2^, the risk of acquiring diabetes is 93 times higher [9]. A startling 50% of new cases of type 2 DM in children are reported in some hospitals [10]. This necessitates understanding the pathogenesis and finding effective strategies to control the global escalation of DM.

In our literature review, we focus on the role of leptin in type 2 DM. Leptin is a peptide hormone that primarily functions to suppress appetite and belongs to a class of hormones known as adiponectin [11]. The peripheral actions of leptin consist of the upregulating proinflammatory cytokines such as tumor necrosis factor-α and interleukin-6 (IL-6), mediators that play an important role in the pathogenesis of type 2 DM and insulin resistance [12].

Leptin is secreted in a pulsatile manner. This pulsatile pattern of leptin release is seen in both obese and lean individuals; however, the amplitude of the release is observed to be higher in obese people [13]. The concentration of leptin correlates to the mass of fat, and an excess of leptin, called leptin resistance, is indicative of obesity [14]. Studies have suggested using serum leptin levels as an indicator of obesity [15]. Contemporary studies have demonstrated that leptin inhibits insulin secretion and has effects opposite to that of insulin on the liver and adipose tissue [16]. This, in essence, means that leptin plays a significant role in the insulin resistance of obesity and type 2 DM.

## Review

Pathophysiology of type 2 diabetes mellitus

In most cases, the pathogenesis of type 2 DM involves a set sequence of events. Initially, there is a state of insulin resistance that causes impaired glucose tolerance. To compensate, the body induces reactive hyperplasia of the beta cells in the pancreas. Eventually, there will be the progressive failure of the beta cells of the pancreas and the development of frank hyperglycemia [17].

The development of obesity is closely linked to the development of insulin resistance and alterations in glucose metabolism via a phenomenon called lipotoxicity [18,19]. With the development of obesity, there is an increase in the levels of free fatty acids (FFAs). There are two main ways by which FFAs contribute to insulin resistance and lipotoxicity. According to Randle et al., increased levels of FFA lead to the buildup of acetyl-CoA and citrate within the muscle which leads to the inhibition of two important enzymes of glycolysis, namely, phosphofructokinase and pyruvate dehydrogenase which further leads to the buildup of glucose and glucose-6-phosphate. Building up glucose and glucose-6-phosphate decreases the insulin-mediated glucose uptake contributing to insulin resistance [20].

FFAs also cause insulin receptor activation and further downstream effects [21]. Two main mechanisms are responsible for this, namely, the impairment of insulin receptor-mediated downstream effects, and the impairment of glucose transporters. According to Dresner et al., FFA impairs the downstream signaling of insulin receptors via an inhibitory effect on phosphoinositol-3-kinase, an important downstream molecule through which the insulin receptor exerts its effects [22]. According to Karnieli and Armoni, in diabetics, there is a reduction in the overall expression of the glucose transporters which is secondary to the depletion of the intracellular pool of receptors. Moreover, inhibition of the full expression of the existing receptors in these subjects has been noted [23].

Owing to all these events, there is a decrease in glucose metabolism and glycogen synthesis by the liver leading to increased glucose in the blood or hyperglycemia [22]. In the initial stages, this rise in blood glucose is counteracted by the compensatory increase in insulin secretion via beta cell hyperplasia. As insulin resistance exists at the tissue level, a vicious cycle of insulin resistance-mediated hyperglycemia occurs, causing a feedback increase in the beta cells, eventually leading to beta-cell failure [24].

Pertaining to our current discussion, leptin plays an important role in the pathophysiology and sequelae of type 2 DM [13]. Leptin is an adipokine produced by adipose tissue. In normal healthy individuals, it suppresses hunger and regulates weight. However, leptin levels are unusually high in obese individuals suggesting resistance to its effects at higher levels, thereby creating a state of increased leptin [25]. Apart from this, studies suggest that leptin creates a state of insulin resistance, which then leads to the state of obesity by altering glucose metabolism [26]. Therefore, this creates a feedback loop in which leptin creates insulin resistance that leads to obesity and obesity causes leptin production eventually leading to beta-cell failure [27].

Role of leptin as a biomarker in body weight regulation

**Figure 1:**
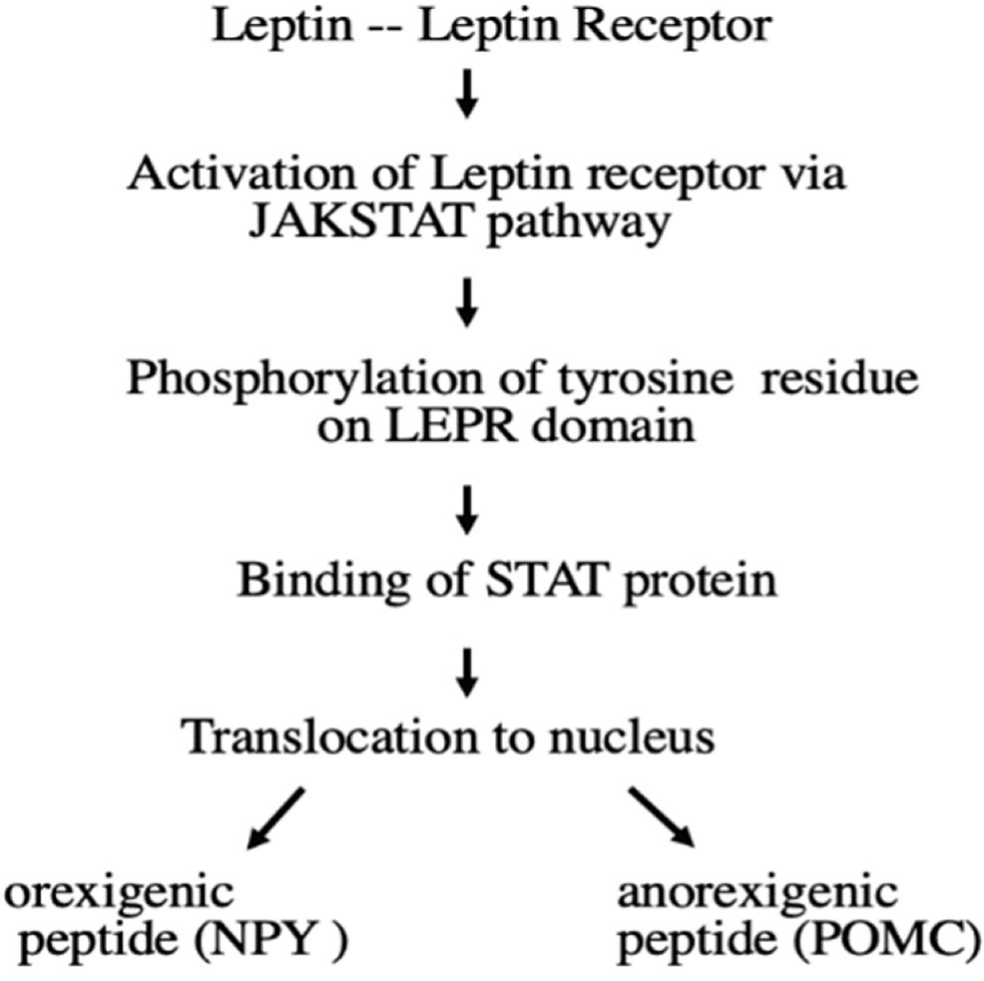
Mechanism of action of leptin. Image credits: Kripa Tiwari. JAKSTAT = Janus kinase signal transducers and activators of transcription; LEPR = leptin receptor; STAT = signal transducer activator of transcription; NPY = neuropeptide Y; POMC = pro-opiomelanocortin

Leptin receptors are encoded by a specific gene known as LEP-R, which is expressed in the brain and peripheral tissues. When leptin binds to its receptor, it gets activated via the Janus kinase 2 (JAK2) pathway which leads to the phosphorylation of two tyrosine residues on the functional LEP-R’s intracellular domain allowing the binding of STAT proteins. Then, it is translocated to the nucleus, where it acts as a transcription factor to regulate the production of orexigenic (neuropeptide Y(NPY)) and anorexigenic peptides (POMC), as shown in Figure 1 [28-30].

Neuropeptide Y

NPY is found in the highest concentration within the hypothalamus, brain stem, and anterior pituitary. In the arcuate nucleus (ARC) of the hypothalamus, NPY neurons are highly expressed. ARC projects to paraventricular nuclei (PVN) and dorsomedial nuclei [31-33]. ARC has a unique anatomic structure, i.e., it lacks a blood-brain barrier. Thus, NPY in ARC serves as a feeding center that can sense and integrate peripheral energy signals, such as blood glucose concentration, ghrelin, leptin, and insulin [34].

NPY exerts its effects through NPY receptors, mainly, Y1, Y2, Y4, and Y6, all of which are G protein-coupled receptors. ARC NPY neurons are activated in response to energy deficiency and greater metabolic demand, such as increased exercise, colds, and pregnancy [35-38]. Energy deficiency and low glucose concentration activate 40% of the NPY neurons. Ghrelin is also an important activator of NPY neurons in the arcuate nucleus.

In contrast, leptin and insulin suppress the NPY neuron in the ARC nucleus. Most ARC neurons expressing leptin receptors also express insulin receptors [39]. Leptin and insulin suppress ghrelin-induced activation of NPY neurons by 30-40% [34]. Studies performed on mice have shown the relationship between leptin and NPY and their effects. ob/ob mouse had a mutation in a single gene of leptin located on chromosome 6, leading to the formation of a non-functional protein. In contrast, fa/fa fat rat/Zucker rat had a mutation at the level of leptin receptors. These mutant mice were hyperphagic with reduced metabolic rates and lower body temperature leading to obesity [40,41].

Stephens et al. and Schwartz et al. showed that systemic or intracerebroventricular (ICV) injection of leptin in ob/ob mice decreases NPY mRNA levels in the ARC with reductions in food intake and body fat weight [29,42]. On the other hand, Cusin et al. showed that leptin reduces NPY levels in the ARC and PVN of lean and fa/fa Zucker rats. Moreover, when leptin receptors were restored in ARC of leptin receptor knockout mice, recovery of satiety function and reduced body weight were noted [43,44].

In summary, these experiments suggest that ARC is the main region for leptin reception, and the use of leptin decreases NPY levels leading to reduced food intake and decreased body fat.

Pro-opiomelanocortin

**Figure 2:**
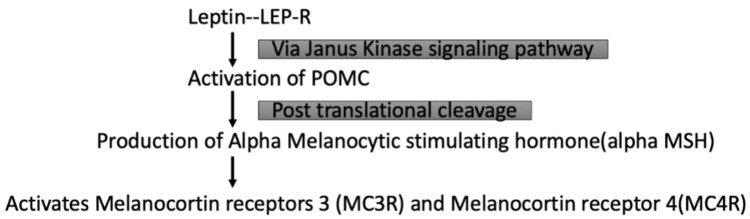
Leptin action via the POMC pathway. Image credits: Kripa Tiwari. LEP-R = leptin receptor; POMC = pro-opiomelanocortin gene

Leptin acts on LEP-R to increase the content of pro-opiomelanocortin (POMC) via the JAK2 signaling pathway, as shown in Figure 2 [45]. Upon activation, POMC undergoes post-translational cleavage to produce an alpha-melanocyte-stimulating hormone (α-MSH) [46]. α-MSH then activates melanocortin receptors 3 (MC3R) and 4 (MC4R) in the hypothalamus, decreasing food intake and increasing energy expenditure [47].

Along with the POMC, another type of neuron that sends peripheral leptin signals to the ARC nucleus is neuropeptide Y/Agouti-related protein (NPY/AgRP), which co-expresses both NPY and AgRP. Both POMC and NPY/AgRP neurons project to cells in PVN that express MC4R, as well as the cells in the ventromedial nucleus that express MC3R [39].

Speakman [39] summarizes the relationship between leptin, GABA, and POMC. High leptin reduces GABA release and NPY/AgRP production but activates the POMC neuron which then releases α-MSH.

AgRP is an antagonist of both MC4R and MC3R receptors, whereas α-MSH is an agonist of MC4R. Hence, increased α-MSH and reduced AgRP levels stimulating MC4R result in two main effects. First, it stimulates the sympathetic system, which releases noradrenaline-expressing beta-adrenergic receptors and stimulates the metabolic rate leading to fat loss. Second, it inhibits food intake.

When there is a negative balance due to decreased food intake and fat loss, leptin production decreases, and there is an opposite effect in the ARC nucleus. NPY/AgRP activation releases more AgRP whereas POMC is inhibited, leading to reduced α-MSH. This leads to decreased sympathetic activity, increased hunger, food-seeking behavior, and hyperphagia [39,48]. Hence, it summarizes the role of leptin in maintaining body weight regulation.

Similarly, in humans, when the leptin hormone is deficient due to a gene mutation, it leads to obesity like in ob/ob mice [49]. Various studies have shown that the defect in leptin receptors also leads to congenital obesity, which is more prevalent in people of European descent but is currently underdiagnosed due to a lack of approach to genetic testing [50].

Similar to leptin, different peripheral and central factors affect food intake, body weight, and energy expenditure, with insulin, corticotropin-releasing factor, and IL-1B being some of them [51,52]. Insulin also acts similarly to leptin. When ICV insulin was injected into normal rats, it was shown to inhibit NPY neurons [53].

Glucoregulatory actions of leptin and leptin levels in type 2 diabetes mellitus

Adipocytes secrete adipocytokines such as leptin and adiponectin, which play a critical role in energy, lipid, and glucose metabolism in the body [54]. Obesity, insulin resistance, and type 2 DM have been linked to an imbalance in these adipocytokine levels [55].

Leptin is a protein released largely by white adipose tissue that regulates food intake, glucose metabolism, and energy expenditure independent of insulin [56,57]. Leptin regulates blood glucose either directly through peripheral tissues or indirectly through the central nervous system (CNS) [58]. Centrally, these effects are mediated via actions on leptin receptors (LepRs) expressed by neurons in the CNS [59,60]. Leptin regulates body weight mainly by GABAergic neurons, whose location is not yet clear, and by neurons within the ventral premammillary nucleus of the hypothalamus [61,62]. On the other hand, the effects of leptin on glucose homeostasis in the context of obesity and insulin resistance are mediated by POMC-expressing neurons within the hypothalamic arcuate nucleus [63,64]. Peripherally, leptin regulates glucose levels by acting directly on its receptor, i.e., leptin receptor (LepRb) located in various peripheral tissues. Among several variants of the leptin receptor gene, the long LepRb isoform is thought to mediate all actions of leptin via the activation of multiple downstream intracellular signaling pathways [65].

Leptin directly modulates the secretion of hormones from the endocrine pancreas. It decreases insulin and glucagon secretion. The underlying mechanisms include activation and membrane translocation of KATP channels, which hyperpolarize the beta-cell membrane, thereby reducing insulin secretion [66].

The liver receives instructions directly from circulating leptin to regulate lipid and glucose metabolism. Leptin impedes gluconeogenesis via insulin receptor substrate-2 and depletes triglyceride content in the liver [67,68]. Leptin administration can also decrease triglyceride content and augment fatty acid oxidation in the liver by directly acting on Kupffer cells [69].

In skeletal muscle, leptin activates AMPK, raises fatty acid uptake, increases fatty acid oxidation, reduces triglyceride formation, and boosts thermogenesis [70-73]. Various, but not all studies, show that exposure of skeletal muscle and muscle cells to leptin augments glucose uptake, glycogenesis, and glucose oxidation [74-76].

**Figure 3:**
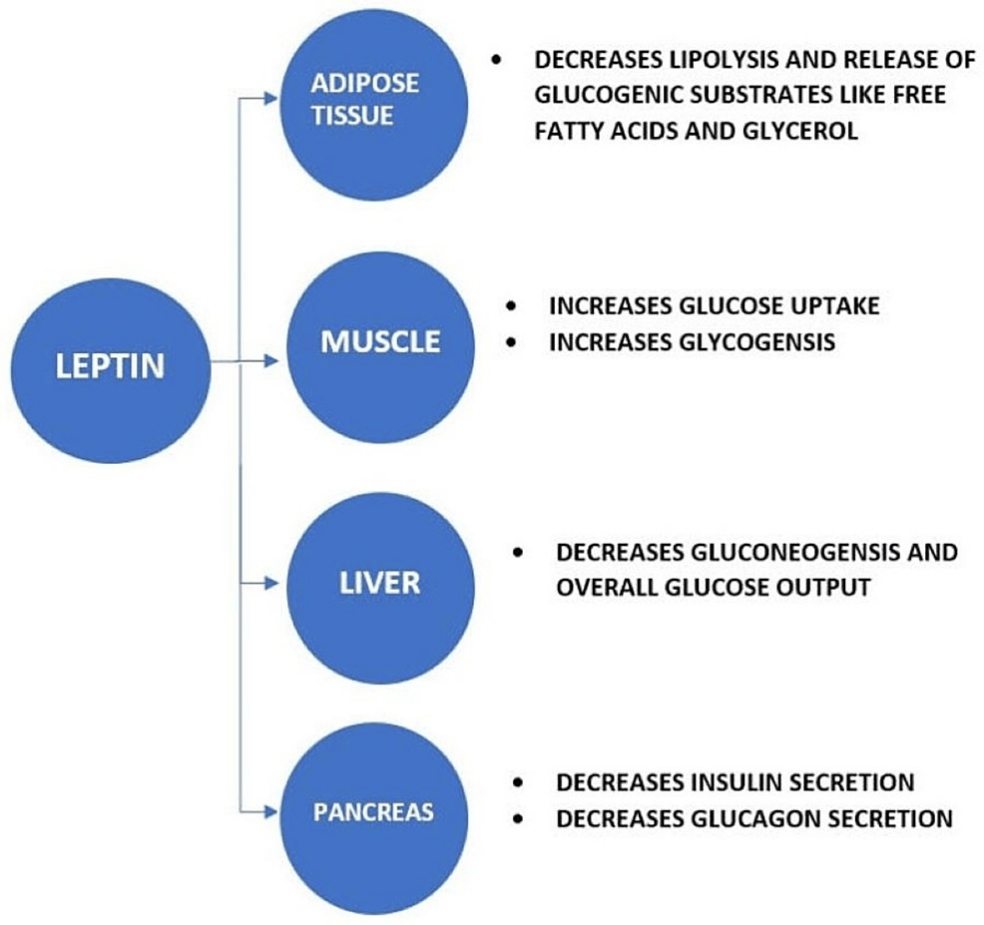
Metabolic actions of leptin. Image credits: Dhriti Patel.

Adipocytes express LepR. Being the main source of leptin, they are exposed to high local leptin concentrations. Leptin inhibits the release of the counter-regulatory hormone corticosterone, which inhibits lipolysis in white adipose tissue, lowering fatty acid and glycerol release, thereby reducing the flux of glucogenic substrates to the liver. It also reduces glucose uptake by the adipocytes [77]. All the above-mentioned glucoregulatory effects of leptin are illustrated in Figure 3 [58,78-80].

Leptin and Appetite

Multiple studies have indicated the presence of leptin receptors in pancreatic beta cells, implying an insulin connection [81,82]. Leptin inhibits insulin secretion from pancreatic cells by binding to leptin receptors [83]. It lowers both basal and glucose-stimulated insulin production via ATP-dependent potassium channels. Leptin reduces cAMP signaling and lowers cAMP levels in response to cell stimulation via the AKT and protein kinase C pathways, as well as decreasing insulin secretion from cells, thereby preventing insulin hypersecretion [84]. Insulin, on the other hand, acts directly on adipocytes to boost leptin synthesis and secretion from white adipose tissue. A rise in blood leptin levels results in less appetite, less food consumption, and weight loss. In humans, congenital leptin deficiency has been associated with severe obesity, glucose intolerance, and insulin resistance [85].

**Table 1:** Leptin and insulin. Table credits: Dhriti Patel. cAMP = cyclic adenosine monophosphate; ATP = adenosine triphosphate

Molecule	Action	Outcome
Leptin	Lowers cAMP levels and inhibits ATP-gated potassium channels	Reduction of insulin secretion
Insulin	Direct action on white adipose tissue to release more leptin	Increase in leptin secretion

By acting on hypothalamic receptors, leptin is a critical signaling molecule in regulating body weight and, as a result, fat formation. It is considered an anti-obesity hormone because it suppresses appetite when the body has sufficient energy stores [86,87]. The mass of adipocytes is directly proportional to serum leptin levels, which, in turn, is inversely related to insulin sensitivity [88]. As a result, the amount of adipose tissue in the body is reflected in leptin secretion and expression [89,90].

As insulin is required for the synthesis and storage of triacylglycerol in fat cells, there is a depletion of body fat stores in type 2 diabetes. The lowering of body fat accumulation causes plasma leptin levels to drop [91]. Type 2 diabetes is characterized by hyperglycemia and hyperphagia, and both insulin and leptin deficiency can explain this. High fasting hyperglycemia is known to affect leptin levels in diabetics with insulin insufficiency, reducing blood levels of leptin [92]. Similarly, lipoatrophy animal models with no adipose tissue are hypoleptinemic and have metabolic abnormalities such as hyperglycemia, insulin resistance, hyperlipidemia, and obesity [93]. It has also been proven that serum leptin levels are inversely related to hemoglobin A1c levels. This shows that glycemia affects serum leptin levels and that hyperglycemia lowers leptin levels [94]. Obese patients typically have high amounts of leptin, which might be due to leptin resistance that is, in turn, caused by deficiencies at or downstream of the leptin receptor, activation of inhibitors of leptin signaling, or changes in leptin transport across the blood-brain barrier [88,95]. Overall, data suggest that leptin signaling in the brain can help normalize diabetic hyperglycemia in people with type 2 diabetes [96].

Impact of various lifestyle modifications on leptin levels

Lifestyle modifications have been shown to affect leptin levels in the human body. These include a multitude of factors, including weight loss, exercise, and reducing dietary fat intake, among others. Leptin levels increase in obesity, and subcutaneous fat has been a major factor in determining circulating leptin levels [97]. Multiple studies have been conducted that aim to assess the effects of weight loss on the levels of circulating leptin, and most of these have shown that a weight reduction subsequently leads to a decrease in the circulating leptin levels, with a predominant fall occurring early after the onset of energy restriction [98-100].

In a study by Edwards et al., there was a significant reduction in the fasting leptin levels from baseline to 5% weight loss (-8.25 ng/mL) and between 5% and 15% weight loss (-1.88 ng/mL) [98]. This study did not assess changes in leptin levels when the weight loss was <5%. However, other studies that assessed this variable reported conflicting findings. Varady et al. found that there was no significant change in circulating leptin levels in women with obesity who lost <5% weight, whereas Keim et al. found that after one week of energy restriction (after only 0.5% weight loss), circulating leptin levels reduced by 57% [100,101]. On the other hand, a study by Magkos et al. revealed a more progressive decrease in circulating leptin levels with a 5%, 11%, and 16% weight loss [102].

Fasting has also been shown to correlate with circulating leptin levels. Energy restriction reduces glucose availability in the bloodstream, reducing insulin levels, which, in turn, decreases the short-term leptin concentration [103-105]. Total fasting for 52 hours in obese (BMI >28 kg/m^2^) and normal weight (BMI <28 kg/m^2^) subjects resulted in a serum leptin levels reduction of 72% and 64%, respectively [104]. According to a study by Wadden et al., energy restriction and weight loss over a longer period (4-40 weeks) can induce reductions in serum leptin levels. The effect of three energy-restricted diets on the leptin concentration in obese female subjects (split into three groups, each with a different diet) was assessed over four weeks, and at the end of the first week, serum leptin levels had reduced significantly in all three groups at week one (up to 66%) and then reduced gradually until week four (up to approximately 80%). During the first week, the changes in energy intake influenced the leptin concentrations more than the changes in body fat mass [106].

Irrespective of the changes in adiposity, short-term energy-restriction diets may lead to fast reductions in serum leptin levels. This, in turn, could potentially result in the reversion of leptin resistance. A more pronounced reduction of serum leptin levels will follow, which is triggered by restricted dietary intake along with loss of body fat mass in the long term [107]. Physical exercise alone (when not accompanied by other lifestyle modifications) has also been shown to reduce circulating leptin levels [108].

Therefore, it is evident that lifestyle modifications significantly impact the circulating serum leptin levels in the human body.

Leptin as a potential target for various treatment strategies

The therapeutic aspects of leptin have been studied for decades now and it certainly plays a valuable role in the treatment of leptin-deficient conditions. Mutations in the leptin gene can lead to specific leptin-deficient conditions such as lipodystrophy syndromes, hypothalamic amenorrhea, anorexia nervosa, and congenital leptin deficiency [109]. The signs and symptoms in such conditions may vary from increased insulin resistance and hyperglycemia to severe endocrine disruptions and morbid obesity [110].

Leptin replacement therapy has been approved by the Food and Drug Administration for use in congenital leptin deficiency and generalized lipodystrophy syndromes [111]. The pharmaceutical form approved known as metreleptin is administered subcutaneously and is known to reverse the metabolic abnormalities seen in these conditions [112]. It leads to substantial decreases in body weight, plasma insulin levels, and blood glucose levels, significantly improving insulin sensitivity [113,114]. A study conducted among nine patients with lipodystrophy and leptin deficiency resulted in an absolute reduction of HbA1c by 1.9%, a reduction in triglyceride level by 60%, and an increase in high-density lipoprotein cholesterol by 30% [114,115].

Historically, there was an assumption that leptin therapy would have a similar potential effect on the treatment of common obesity, also referred to as diet-induced obesity [116]. It was eventually established that the majority of these obese individuals do not have decreased circulating levels of leptin. Rather, metreleptin plays a limited role in the treatment of common obesity due to the presence of high leptin levels in the circulation of these patients [117]. Such obesity-induced hyperleptinemia contributes to central leptin resistance and such patients do not respond to exogenously administered leptin [118].

It is important to note that in the absence of any stimulating factors, such as a high-fat diet, hyperleptinemia in transgenic lean mice is known to improve insulin sensitivity and glucose metabolism [119]. However, when exposed to a high-fat diet, hyperleptinemia promotes leptin resistance [120]. In the context of obesity-related leptin-resistant conditions, an approach toward a partial leptin reduction state in the hypothalamus can restore the sensitivity of both insulin and leptin, a therapeutic intervention that directly or indirectly inhibits leptin levels and triggers significant weight loss along with metabolic homeostasis [121].

Leptin therapy in animals has been shown to improve blood glucose levels by decreasing hepatic glucose production, inhibiting glucagon secretion, and increasing glucose uptake [122]. Clinical trials conducted recently in patients with type 1 DM have shown that administering leptin adjuvant to insulin therapy leads to 3.7% and 6.6% weight loss by weeks 12 and 20, respectively (with a p-value of 0.003) [123]. Moreover, insulin dose requirements were significantly reduced in such patients, by 12.6% and 15.0% at weeks 12 and 20, respectively (with a p-value of 0.006), due to improved insulin sensitivity [123,124]. Another positive outcome of the study was the absence of any serious adverse events due to the administration of subcutaneous leptin. However, the therapy did not prove to be efficacious in improving glycemic control as there was no statistically significant change in HbA1c levels after 20 weeks compared to the baseline value (p-value was 0.75) [124].

Patients with type 2 DM who are non-obese with normal or low leptin levels may also benefit from this approach as they are more leptin-sensitive than obese patients [44]. Data from recent clinical trials conducted among obese patients with type 2 DM indicated that leptin therapy is ineffective or only marginally effective in improving metabolic abnormalities and insulin resistance [125]. The studies also revealed that body weight and inflammatory markers remained unaltered in hyperleptinemic obese and diabetic patients treated with metreleptin [126]. The results were statistically significant with no change in BMI or fat mass (% body weight) after two weeks of treatment with low dose (30 mg/day) and high dose (80 mg/day) leptin [125]. These studies indicate a close association of obesity with resistance to the metabolic effects of leptin.

It is difficult to precisely describe the mechanisms involved in leptin resistance but various possibilities have been hypothesized. Mutations at the level of the blood-brain barrier transport proteins or leptin receptors can impair the intracellular signaling of leptin [127]. Strategies that improve leptin pharmacokinetics and increase its transport through the blood-brain barrier, independent of the leptin transporter, constitute one of the potential therapeutic targets [128]. Epigenetic regulation, specifically DNA methylation of the leptin promoter, is another mechanism that has been studied in detail [129]. Obese adolescents with insulin resistance were found to have an inverse relationship with the methylation frequency of the leptin promoter gene, determined by the methylation-specific polymerase chain reaction in DNA obtained from peripheral blood samples [130]. Another study showed that hypermethylation of the POMC promoter gene and hypomethylation of the NPY promoter gene interferes with the binding of transcription factors, and blocks the effect of high leptin levels, thus leading to obesity [131,132]. These epigenetic regulations are dynamic and can be altered by various environmental factors and lifestyle modifications. As described in a recent review, an epigenetic diet comprises various minerals, vitamins, polyphenols, and phytochemicals that have the potential to modify gene expression [133]. However, most of the research to date has emphasized the effect of these nutrients on cancer genes. The epigenetic markers involved in leptin resistance serve as therapeutic targets for obesity and its related comorbidities. Therefore, interventions targeted at the design of leptin sensitizers or reduction of leptin levels in the circulation can potentially improve leptin, as well as insulin sensitivity, along with antidiabetic agents.

Various pharmacological therapies have been employed to overcome leptin resistance in the past, including leptin administration in combination with amylin, glucagon-like peptide 1 (GLP-1), and fibroblast growth factor 21 (FGF-21) [122]. However, to achieve an optimal level of leptin responsiveness, these therapies required a significant amount of weight loss with lifestyle modifications [134]. A potential approach in this scenario is a combination therapy of partial leptin reduction (e.g., monoclonal leptin-neutralizing antibodies) along with GLP-1 agonists, FGF-21, or even insulin [121]. As one of the most common side effects of insulin treatment remains significant weight gain, combining it with leptin-neutralizing antibodies would restore leptin sensitivity and prove beneficial for the management of obesity in type 2 DM [135].

## Conclusions

Our review aimed to better understand the role of leptin in type 2 DM and summarize most of the relevant literature. The approach toward managing type 2 DM should be multifaceted as DM is a multisystemic disease. Undoubtedly, massive efforts have been made in the therapeutic arena for type 2 DM, but as a condition that is steadily on the rise and posing a major public health concern, we recommend further studies to explore other hormonal factors that play a role in the pathophysiology of type 2 DM. Leptin is a potential promising therapeutic target for type 2 DM. For a condition as extraordinary as type 2 DM, both in terms of people affected and its impact on quality of life, it is imperative we explore every avenue to reduce the burden of this disease.
